# Metabolic and Lipidomic Profiling of Vegetable Juices Fermented with Various Probiotics

**DOI:** 10.3390/biom10050725

**Published:** 2020-05-06

**Authors:** Hyuk-Jin Chung, Hwanhui Lee, Guknam Na, Heechul Jung, Dong-Gun Kim, Sang-Ick Shin, Seong-Eun Jung, Il-dong Choi, Jae-Hwan Lee, Jae-Hun Sim, Hyung-Kyoon Choi

**Affiliations:** 1 College of Pharmacy, Chung-Ang University, Seoul 06974, Korea; hjchung0812@gmail.com (H.-J.C.); hwanhui56@gmail.com (H.L.); 2 Korea Yakult Co., Ltd., Yongin 17086, Korea; biongn@re.yakult.co.kr (G.N.); yk58jhc@re.yakult.co.kr (H.J.); kimdg@re.yakult.co.kr (D.-G.K.); muse123@re.yakult.co.kr (S.-I.S.); krus00@re.yakult.co.kr (S.-E.J.); cid1010@re.yakult.co.kr (I.-d.C.); jaehwan@re.yakult.co.kr (J.-H.L.); jhsim@re.yakult.co.kr (J.-H.S.)

**Keywords:** vegetable juice fermented with probiotics, *Lactobacillus*, *Bifidobacterium*, metabolic profiling, lipidomic profiling

## Abstract

Fermented vegetable juices have gained attention due to their various beneficial effects on human health. In this study, we employed gas chromatography–mass spectrometry, direct infusion-mass spectrometry, and liquid chromatography–mass spectrometry to identify useful metabolites, lipids, and carotenoids in vegetable juice (VJ) fermented with *Lactobacillus plantarum* HY7712, *Lactobacillus plantarum* HY7715, *Lactobacillus helveticus* HY7801, and *Bifidobacterium animalis* ssp. *lactis* HY8002. A total of 41 metabolites, 24 lipids, and 4 carotenoids were detected in the fermented and non-fermented VJ (control). The lycopene, α-carotene, and β-carotene levels were higher in VJ fermented with *L. plantarum strains* (HY7712 and HY7715) than in the control. Proline content was also elevated in VJ fermented with HY7715. Uracil, succinic acid, and α-carotene concentration was increased in VJ fermented with HY7801, while glycine and lycopene levels were raised in VJ fermented with HY8002. This study confirmed that each probiotic strain has distinctive characteristics and produces unique changes to metabolic profiles of VJ during fermentation. Our results suggest that probiotic-fermented VJ is a promising functional beverage that contains more beneficial metabolites and carotenoids than commercial non-fermented VJ.

## 1. Introduction

The World Health Organization (WHO) defines probiotics as living microorganisms that, when consumed at a sufficient level, promote host health [1]. Representatively, *Lactobacillus acidophilus, B. lactis*, *Enterococcus faecalis*, *Enterococcus faecium*, *Lactococcus lactis*, and *Streptococcus thermophilus* are probiotic strains that produce lactic acid. *Bacillus* and *Saccharomyces* strains, which do not produce lactic acid, are also considered probiotics [2]. Traditional probiotic-fermented foods, such as yogurt, cheese, miso, and kimchi, are commonly consumed for their health benefits [3]. Consuming probiotics is clinically proven to reduce symptoms related to imbalanced gut microbiota, abnormal immune system response related to cold and influenza, cardiovascular disease, and gastrointestinal discomfort [4].

Vegetable juices (VJs) have gained widespread popularity as an alternative to raw vegetables and fruits to supply micronutrients, phenolic compounds, carotenoids, and fiber [5,6]. A diet rich in vegetables may reduce the risk of cardiovascular disease, protect from oxidative stress, and prevent some types of cancer [7,8,9]. Multiple studies have suggested the beneficial effects of fermented vegetables and VJs. *Lactobacillus*, *Lactococcus*, and *Enterococcus* strains are present in traditional fermented health foods, such as sauerkraut, pickled cucumbers, and kimchi [10], and consuming fermented Asian vegetables is an easy way to boost probiotic intake [11]. Strawberry, onion, and tomato juices fermented by *Lactobacillus*, *Lactococcus*, *Leuconostoc,* and *Saccharomyces* display antioxidant capacity compared to non-fermented vegetable juices [12]. The antioxidant properties of orange and carrot juices are enhanced following fermentation by two *Bifidobacterium* strains [6]. In addition, kale juice fermented by different *Lactobacillus* strains is abundant in calcium, phosphorus, and magnesium, and those fermented with *L. acidophilus* IFO 3025 and *L. brevis* FSB-1 display improved nutritional and mineral composition, respectively [13]. The color and level of volatile compounds in fruit and VJs are also known to change after fermentation [12].

Metabolomics and lipidomics can be used to investigate the metabolic and lipidomic changes in vegetables and VJ during fermentation. Recently, it was reported that nuclear magnetic resonance spectrometry (NMR) and liquid chromatography–mass spectrometry (LC-MS) can rapidly discriminate the metabolic profiles of vegetable juice and medium fermented with different *Lactobacillus* strains [14,15]. This suggests that different lactic acid bacteria have unique characteristics that influence the types of metabolites produced, and multivariate data analysis can be used to assess the metabolic changes following fermentation. Filannino et al., who analyzed vegetable and fruit juices using LC and gas chromatography–mass spectrometry (GC-MS), found that the levels of malic acid, branched-chain amino acids, and gamma-aminobutyric acid were altered following fermentation [16]. Tomita et al., who subjected 45 metabolites and 62 volatile compounds in sunki (a fermented pickle from Japan) to NMR and GC-MS analysis, reported that acetic acid concentration was positively correlated with pH and negatively correlated with lactate and ethanol levels [17]. In another study, metabolites in fermented ginseng extracts were analyzed using GC-MS and an electronic tongue [18]. Ginseng extracts fermented by four different starter cultures could be distinguished according to their sugar and organic acid content, as well as their taste.

In this study, we hypothesized that each probiotic strain is able to generate distinct metabolic and lipidomic profiles during fermentation of VJ. To test this hypothesis, we used GC-MS, direct infusion-mass spectrometry (DI-MS), and LC-MS to assess the relative levels of various metabolites and intact lipid species in VJs fermented by four different probiotic strains: *Lactobacillus plantarum* HY7712, *Lactobacillus plantarum* HY7715, *Lactobacillus helveticus* HY7801, and *Bifidobacterium animalis* ssp. *lactis* HY8002. Several beneficial metabolites and lipid species, including carotenoids, were discovered in fermented VJ, which could have practical implications for improving public health.

## 2. Materials and Methods

### 2.1. Probiotic Cultures and Vegetable Juice Fermentation

The VJ and fermented VJs were provided by Korea Yakult Co., Ltd. (Yongin, Korea). The juice consisted of tap water, 18–24% organic carrot juice concentrate (Ernteband, Winnenden, Germany), 7–10% organic tomato paste (Attianese, Naples, Italy), 1–3% organic mixed VJ 1 (lettuce 41%, celery 32%, spinach 27%) (MSC, Gyeongsangnam-do, Korea), 0.01–1% organic broccoli juice (MSC, Gyeongsangnam-do, Korea), 0.01–1% organic zucchini and pumpkin juice concentrate (Ernteband, Winnenden, Germany), and 0.01–1% organic mixed VJ 2 (bok choy 22%, tatsoi 21%, lettuce 20%, broccoli leaf 11%, crown daisy 11%, curled mustard leaf 8%, chard 7%) (MSC, Gyeongsangnam-do, Republic of Korea). The juice composition was based on “Haru Yache Original” from Korea Yakult Co., Ltd. *Lactobacillus* strains HY7712, HY7715, and HY7801, and *Bifidobacterium* HY8002, from the Korea Yakult Probiotics Library (Yongin, Korea), were cultured in De Man, Rogosa and Sharpe (MRS) media (BD Difco, Maryland, USA) before each strain (>10^7^ CFU/mL) was inoculated into a sample of the sterilized VJ (30 min, 100 °C). Non-inoculated VJ (pH 4.8) was considered as the control. The inoculated juices were fermented at 37 °C for 24 h (The 24 h-fermented VJs with HY7712: 1.5 × 10^9^ CFU/mL, pH 3.8; HY7715 : 1.0 × 10^9^ CFU/mL, pH 3.8; HY7801 : 1.1 × 10^8^ CFU/mL, pH 3.6; HY8002 : 7.9 × 10^8^ CFU/mL, pH 4.1), then stored at −70 °C until analysis.

### 2.2. Comprehensive Metabolic Profiling Using GC-MS

The fermented and non-fermented juices (100 µL) were transferred to separate microfuge tubes (Eppendorf, Hamburg, Germany), and extracted with 1 mL of methanol (HPLC grade; Fisher Scientific, Pittsburgh, PA, USA). The samples were briefly vortexed and sonicated for 30 min at 40 kHz to improve extraction yields [19]. After sonication, the samples were centrifuged at 1000× *g* for 3 min at 4 °C, and the supernatant was filtered through a 0.45 μm polytetrafluoroethylene (PTFE) syringe filter (Whatman, Maidstone, UK). Of this, 200 µL were transferred to GC vials and dried under nitrogen for 20 min. The derivatization and GC-MS analysis of each sample were conducted according to previously reported methods [20]. A split ratio of 1:15 was used, and the detector voltage was set to 1153 V. The oven temperature was set at 60 °C and programmed to increase to 185 °C at 5 °C/min (hold time 3 min), then to 205 °C at 3 °C/min, and finally to 310 °C at 5 °C/min.

### 2.3. Comprehensive Lipid Profiling Using DI-MS

The fermented and non-fermented juices (50 µL) were transferred to separate microfuge tubes, and intact lipid species were extracted using the modified Matyash methyl tert-butyl ether (MTBE) method [21,22]. Briefly, 1 mL of MTBE (Sigma-Aldrich, St. Louis, MO, USA), 300 µL of methanol, and 10 µL of phosphatidylethanolamine (PE) 17:0/17:0 as an internal standard were added and vortexed. The sample was incubated for 1 h with shaking at room temperature. Two hundred fifty microliters of water (HPLC grade, Fisher Scientific, Pittsburg, PA) was added to sample for phase separation, and then the mixture was centrifuged at 1000× *g* for 10 min. The upper phase was collected and dried under nitrogen gas. The dried lipid extract was dissolved in 300 µL of chloroform/methanol (2:1, *v*/*v*) solution. For DI-MS analysis, methanol/chloroform (9:1, *v*/*v*) containing 7.5 mM ammonium acetate solution was added to each lipid extract. DI-MS analysis of each sample was performed as previously reported [22]. A linear ion-trap mass spectrometer (LTQ-XL, Thermo Fisher Scientific, San Jose, CA, USA) coupled with an automated nanoelectrospray system (Triversa NanoMate System, Advion Biosciences, Ithaca, NY, USA) was used in positive- and negative-ion modes. The lipid extract was analyzed in full scan mode for 2 min, and the scan range was set at *m*/*z* 400–1200 and 500–1300 in positive and negative mode, respectively. Mass spectra were acquired in both positive mode (capillary voltage of 45 V, tube lens voltage of 95 V) and negative mode (capillary voltage of −45 V, tube lens voltage of −95 V). Tendem MS spectra was obtained to pooled samples from each group to identify lipid species. Lipid species were identified by comparing LipidBlast database by Kind et al. [23]. In addition, the in-house MS/MS library and Lipidmaps database [24] were used for identification.

### 2.4. Carotenoid Analysis Using LC-MS

The fermented and non-fermented juices (50 µL) were transferred to separate microfuge tubes and extracted with 360 μL of acetone (HPLC grade; Burdick & Jackson, Musketon, MI, USA) containing 0.1% butylated hydroxytoluene (BHT; Sigma-Aldrich, St. Louis, MO) and 540 μL of hexane (HPLC grade, Burdick & Jackson) containing 0.1% BHT. The mixture was briefly vortexed, sonicated for 5 min at 4 °C, then centrifuged at 1000× *g* for 10 min at 4 °C. After centrifugation, the supernatant was collected into microfuge tubes, and the residue re-extracted with 360 μL of acetone and 540 μL of hexane. The mixture was vortexed, sonicated, and centrifuged as described above. The supernatant was collected into the microfuge tubes used for the first extraction, and 200 μL of water (HPLC grade; Fisher Scientific) with 0.1% BHT was added for phase separation. The top phase was collected and filtered through a 0.2 μm PTFE syringe filter (Whatman). The filtrate was transferred into an amber vial, and 2 μL of β-apo-8′-carotenal was added (Sigma-Aldrich, St. Louis, MO, USA), with 100 μg/mL used as an internal standard. Each sample was dried under nitrogen for 20 min and resuspended in 100 μL of acetonitrile (HPLC grade; Fisher Scientific) and methanol solution (7:3, *v*/*v*). 

To increase the stability of the carotenoid standard solutions, a 100 μg/mL stock solution was prepared in hexane with 0.1% BHT [25]. The standard mixture was prepared with 100 μg/mL each of α-carotene, β-carotene, and lycopene. Lutein (20 μg/mL) was added to the stock solution before the mixture was dried under nitrogen, and then resuspended in 100 μL of acetonitrile:methanol solution (7:3 *v*/*v*). LC-MS analysis of each sample was conducted as previously reported, using an Accela LC (Thermo Fisher Scientific, San Jose, CA, USA) equipped with a degasser, Accela 600 pump, linear ion-trap mass spectrometer (LTQ-XL, Thermo Fisher Scientific), and Accela AS autosampler [26]. A 1.9 µm Hypersil Gold column (Part no. 25002-102130; 100 mm × 2.1 mm; Thermo Scientific, San Jose, CA, USA) was used, and the column oven temperature was 35 °C. The autosampler tray temperature was 10 °C, and the elution flow rate was 300 μL/min. Water with 0.1% formic acid and a mixture of acetonitrile, methanol, and MTBE (70:20:10) with 0.1% formic acid served as solvents A and B, respectively. The gradient was set to 25% solvent A and 75% solvent B and maintained for 2 min. At 5 min, solvent B was increased to 98% and maintained for 17 min. After each run, the equilibrium time was 3 min with 25% solvent A and 75% solvent B. The carotenoids in the fermented and non-fermented juice were identified by comparing the retention times and MS/MS spectra with those of corresponding carotenoid standards. Additionally, the control and fermented juices were analyzed on the micro-LC-LTQ-Orbitrap-XL instrument (Thermo Fisher Scientific) to identify the carotenoids by means of exact mass measurements and isotope patterns.

### 2.5. Statistical Analysis

The GC-MS, DI-MS, and LC-MS data were collected in Microsoft Office Excel (version 2016; Microsoft, Redmond, WA, USA) and used for principal component analysis (PCA), partial least squares–discriminant analysis (PLS-DA), and pathway analysis. The differences in the relative levels of metabolites, lipids, and carotenoids were evaluated by Mann–Whitney test in SPSS software (version 23; IBM, Somers, NY, USA), and those with *p* < 0.05 were considered statistically significant. For PCA and PLS-DA, all data were mean-centered and scaled to unit variance in SIMCA-P+ software (version 13.0; Umetrics, Umeå, Sweden). Pathway analysis was performed in the web-based software tool MetaboAnalyst (version 4.0) [27].

## 3. Results

### 3.1. Identification and Quantification of Metabolites and Lipids in Fermented and Non-Fermented VJs Using GC-MS, DI-MS, and LC-MS

Comprehensive GC-MS analysis of the fermented and non-fermented VJs identified 41 metabolites: 13 amino acids (β-alanine, γ-aminobutanoic acid, alanine, asparagine, aspartic acid, glutamic acid, glycine, isoleucine, proline, pyroglutamic acid, serine, threonine, and valine), 4 fatty acids (1-monopalmitin, linoleic acid, palmitic acid, and stearic acid), 8 organic acids (acetic acid, citric acid, fumaric acid, lactic acid, malic acid, malonic acid, succinic acid, and tartaric acid), 7 sugars (fructose, galactose, glucose, glucose-6-phosphate, sedoheptulose, sucrose, and xylose), 2 sugar acids (glyceric acid and threonic acid), 5 sugar alcohols (erythritol, glycerol, mannitol, myo-inositol, and xylitol), phosphoric acid, and uracil (Table 1). The levels of lactic acid and succinic acid were significantly higher, whereas those of β-alanine, asparagine, aspartic acid, pyroglutamic acid, serine, linoleic acid, fumaric acid, malic acid, tartaric acid, glucose, glyceric acid, erythritol, and phosphoric acid were significantly lower in VJ fermented with any of the four probiotics than in the control. In VJ fermented with *L. plantarum* HY7712, the levels of one fatty acid (stearic acid), two organic acids (lactic acid and succinic acid), and one alcohol (glycerol) were significantly higher, whereas those of other 28 metabolites were significantly lower, than in the control. In VJ fermented with *L. plantarum* HY7715, the concentrations of one amino acid (proline), two organic acids (lactic acid, succinic acid) and one sugar alcohol (glycerol) were significantly higher, whereas those of 23 other metabolites were significantly lower than in the control. Compared to non-fermented juice, VJ fermented with *L. helveticus* HY7801 had a higher content of two organic acids (lactic acid and succinic acid) and uracil and a lower content of 26 other metabolites. In VJ fermented with *B. lactis* HY8002, the levels of one amino acid (glycine), two organic acids (lactic acid and succinic acid), two sugars (glucose-6-phosphate and xylose), and one sugar alcohol (glycerol) were significantly higher, whereas those of 13 other metabolites were significantly lower, than in the control.

DI-MS analysis detected the following intact lipid species in the fermented and non-fermented VJs: three monogalactosyldiacylglycerols (MGDG; 18:2/18:3, 18:2/18:2, and 18:1/18:2), three lysophosphatidylcholines (Lyso-PC; 18:2, 18:1, and 22:5), one phosphatidylcholine (PC; 18:2/18:2), one phosphatidylethanolamine (PE; 16:0/20:0), and five triacylglycerides (TG; 16:0/18:2/18:2, 18:2/18:2/18:3, 18:2/18:2/18:2, 18:1/18:2/18:2, and 18:1/18:1/18:2) in positive ion mode, and two phosphatidic acids (PA; 16:0/18:2 and 18:2/18:2), four phosphatidylethanolamines (PE; 16:0/18:2, 18:2/18:2, 18:1/18:2, and 18:0/18:2), one phosphatidylglycerol (PG; 16:0/18:2), two phosphatidylserines (PS; 18:2/20:0 and 18:2/22:0), and two phosphatidylinositols (PI; 16:0/18:2 and 16:0/18:1) in negative ion mode (Table 2). In VJ fermented with *L. plantarum* HY7712, the concentration of PE 18:2/18:2, PG 16:0/18:2, PS 18:2/22:0, and PI 16:0/18:1 was significantly higher, whereas that of PE 18:0/18:2 was significantly lower, than in the control. In VJ inoculated with *L. plantarum* HY7715, the levels of PE 18:2/18:2, PS 18:2/22:0, and PI 16:0/18:1 were significantly higher, while those of PE 16:0/18:2 and 18:0/18:2 were significantly lower, than in the control. In VJ fermented with *L. helveticus* HY7801, the levels of PE 18:2/18:2, PE 18:1/18:2, PG 16:0/18:2, PS 18:2/22:0, and PI 16:0/18:1 were significantly higher, while those of PE 18:0/18:2 and PS 18:2/20:0 were significantly lower, than in non-fermented juice. VJ fermented with *B. lactis* HY8002 had a significantly higher content of PE 18:2/18:2, PS 18:2/22:0, and PI 16:0/18:1 and a significantly lower content of Lyso-PC 18:2 and PE 18:0/18:2 than in the control.

Relative levels of carotenoids in the different VJ samples are listed in Table 3. The concentration of lycopene, α-carotene, and β-carotene was significantly higher in VJ fermented with *L. plantarum* HY7712 and HY7715 than in the control. VJ fermented with *L. helveticus* HY7801 had lower levels of lutein and higher levels of α-carotene, while VJ fermented with *B. lactis* HY8002 had significantly higher levels of lycopene, than in the control. 

### 3.2. Probiotic Fermentation of VJ Alters its Metabolic and Lipidomic Profiles

**Figure 1:**
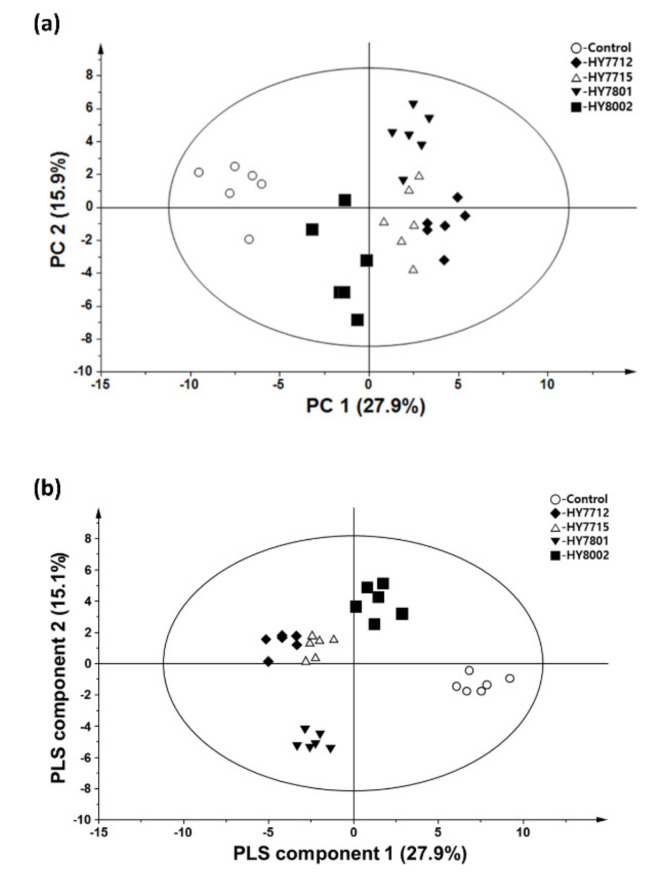
Metabolic and lipidomic data for the fermented and non-fermented VJs. (**a**) PCA score plot. (**b**) Partial least squares–discriminant analysis (PLS-DA) score plot. *n* = 6 in each group.

The metabolic and lipidomic data for the different juices were clearly distinguished in the PCA and PLS-DA score plots Figure 1. The metabolic and lipidomic profiles of VJs fermented by the two *L. plantarum* strains (HY7712 and HY7715) were similar and clearly distinguishable from those of VJ fermented by *L. helveticus* HY7801. Moreover, the data for *B. lactis* HY8002-fermented juice differed from those for *Lactobacillus* HY7712, HY7715, and HY7801-fermented juices. 

**Figure 2:**
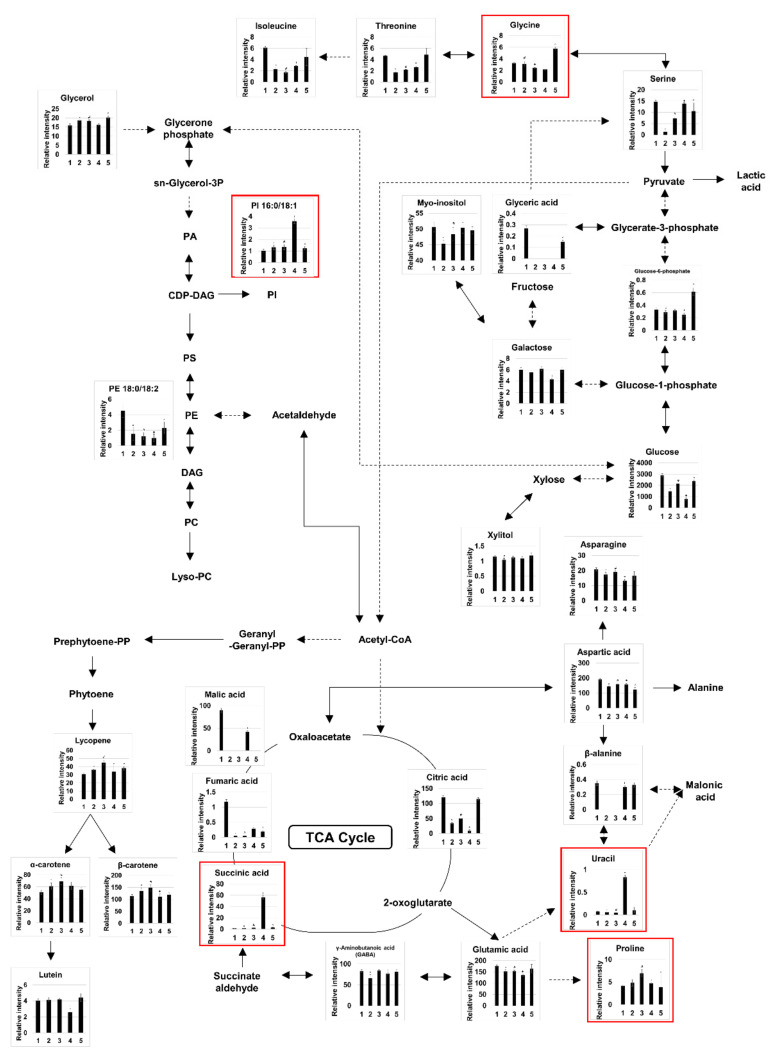
Relative intensity of metabolites, lipids, and carotenoids in the fermented and non-fermented VJs and the related metabolic pathways. Citrate cycle (TCA cycle) and the alanine, aspartate, and glutamate metabolism pathways. Glycine, serine, and threonine metabolism and the glycolysis pathways. Glycerophospholipid metabolism pathway. Carotenoid biosynthetic pathway. Significant differences (*p* < 0.05) between fermented VJs and the control (1) are indicated as follows: ({(2) *L. plantarum* (HY7712), *; (3) *L. plantarum* (HY7715), #; (4) *L. helveticus* (HY7801), +; (5) *B. lactis* (HY8002), ^}, *p* < 0.05). PC, phosphatidylcholine; PE, phosphatidylethanolamine; PI, phosphatidylinositol; PS, phosphatidylserine; PA, phosphatidic acid; DAG, diacylglycerol; CDP-DAG, cytidine diphosphate-diacylglycerol. The major differential compounds (4 metabolites and 1 intact lipid) between the fermented and non-fermented VJs are shown in red.

Our findings confirm that the metabolites and lipids present in fermented VJ differ depending on which probiotic strain was used to produce it. It follows that different probiotics might utilize different nutritional compounds of the juice during fermentation. A metabolic pathway analysis of the 41 metabolites identified by GC-MS showed that the following processes were activated after probiotic fermentation of VJ: alanine, aspartate, and glutamate metabolism; glycine, serine, and threonine metabolism; the citrate cycle; aminoacyl-tRNA biosynthesis; starch and sucrose metabolism; and arginine and proline metabolism (Table 4). The main metabolites of these pathways were largely consistent with the 27 metabolites with a VIP (Variable Importance in the Projection) score above 1.0 in the PLS-DA model (Table 5). The relative abundance of the altered metabolites and lipids and the related metabolic pathways are presented in Figure 2.

## 4. Discussion

We hypothesized that the change in the levels of metabolites, lipids, and carotenoids in VJ might be affected by the activities of probiotic enzymes. Galactosidases, glucosidase, lipase, and leucine aminopeptidase are present in human *Bifidobacteria* [28], while β-galactosidase and β-d-phosphogalactoside galactohydrolase were detected in *Lactobacillus* strains [29]. Malolactic enzymes purified from *L. plantarum* convert l-malate to l-lactate [30]. In addition, the production of exopolysaccharide by *L. rhamnosus* and *L. sakei* is associated with a number of enzymes, including α-d-glucosidase, β-d-glucuronidase, and α-phosphoglucomutase [31,32].

Our results suggest that the production of certain metabolites is markedly enhanced because of the activation of multiple metabolic pathways (Figure 2). In HY7801-fermented VJ, the level of uracil was higher by 11.1 times than in the control. Uracil is a pyrimidine nucleobase that binds to adenine in RNA [33] and is essential for the growth of lactobacilli [34]. *Orostachys japonicus* A. Berger, commonly called rock pine, fermented with *L. plantarum* displays 4.73 times the uracil content of non-fermented *Orostachys japonicus* A. Berger [35], and it is thought that uracil affects the growth and antibacterial activity of this probiotic strain [36]. Uracil and glycerol in soymilk fermented with *L. plantarum* show antihypertensive effects [37]. Moreover, uracil could play an important role in the detoxification of carcinogens such as tobacco smoke [38]. Kim et al. [39] suggested that the anti-inflammatory effects of uracil in garlic might be mediated by modulating NF-κB signaling, and Zimin et al. [40] revealed that acid derivatives of uracil might also exhibit anti-inflammatory properties. We surmised that the anti-inflammatory effects of uracil could be related to the findings of previous studies on *L. helveticus* HY7801. Research in animal models has shown *L. helveticus* HY7801 to have a number of anti-inflammatory effects. Orally administered HY7801 regulates immune biomarkers, including TNF-α, IFN-γ, IL-17A, IL-10, and IL-12, and might be useful in the treatment of rheumatoid arthritis [41]. In addition, oral administration of HY7801 improves vulvovaginal candidiasis by inhibiting the survival of *Candida albicans* and down-regulating TNF-α, COX-2, iNOS, and IL-1β levels [42]. According to Hong et al. [43], administration of HY7801 to mice with colitis altered their intestinal microbiota and fecal metabolite levels.

In HY7801-fermented VJ, the level of succinic acid was also higher by 36.67 times than in the control. Succinic acid is an intermediate of the tricarboxylic acid (TCA) cycle and the end-product of anaerobic fermentation [44] and is widely used in the agricultural, food, and pharmaceutical industries [45]. *L. helveticus* produces succinic acids through citric acid metabolism, which are thought to contribute to the flavor profiles of Emmental and cheddar cheese [46]. In addition, *Streptococcus lactis* (recently *Lactococcus lactis*) converts fumaric acid to succinic acid [47]. Succinic acid production by lactic acid bacteria could be a response to stalled growth resulting from nutrient depletion [48]. Therefore, it is possible that decreasing nutrient levels during VJ fermentation halted the growth of HY7801, thereby stimulating the production of succinic acid. Succinic acid has numerous beneficial effects on human health. Consumption of succinic acid improves nerve cell function [49] and is therapeutic in patients with brain damage [50]. Furthermore, succinic acid is a proposed treatment for cervical cancer [51] and might have antioxidant capabilities [52].

In addition, in HY7801-fermented VJ, the level of PI 16:0/18:1 was higher by 3.54 times than in the control. PI is produced from CDP-diacylglycerol and plays important roles in cell signaling, cell wall structure, and protein metabolism [53,54]. Increased PI production by *Saccharomyces cerevisiae* might be a response to nutrient exhaustion and entry into the stationary phase [53]. Ethanol production by *Saccharomyces* strains is affected by increased PI levels [55]. We speculate that the growth of HY7801 might have been halted because of the depletion of nutrients required for fermentation. Dietary PI supplementation shows promise in the treatment of various diseases, such as diabetic neuropathy [56]. Küllenberg et al. [57] suggested that consumption of phospholipids could have a positive effect on inflammation, cancer, cardiovascular diseases, and liver disease. Notably, oral administration of PI increases the level of high-density lipoprotein-cholesterol (HDL-C) in human plasma [58].

Moreover, proline content of HY7715-fermented VJ was 1.66 times higher than that of non-fermented juice. Proline is an amino acid that participates in protein synthesis [59,60,61]. Its production is upregulated in plants in response to various stresses, including temperature and reactive oxygen species [62], and *Saccharomyces cerevisiae* might synthesize it to adapt to stress during fermentation [63]. Proline production could be related to changes in glutamic acid levels during *Kurthia catenaforma* fermentation [64]. Proline is an essential amino acid that participates in collagen synthesis [65] and is useful for intestinal health [66].

Glycine content of HY8002-fermented VJ was also 1.76 times higher than that of non-fermented juice. Glycine is essential for the formation of secondary protein structure [67]. Moreover, proliferating lactic acid bacteria increase the capacity for glycine production in an *L. salivarius* and *P. acidilactici* co-culture [68]. Glycine consumption reportedly reduces fatigue [69] and prevents skin cancer in animal models [70].

Carotenoids are a group of bioactive tetraterpenoids that exhibit antioxidant properties [71]. Among the carotenoids, lycopene has a role in the prevention of cancer and cardiovascular disease [72], while β-carotene is necessary for the maintenance of skin and mucous membranes, and visual adaptation to the dark. The latter is a functional ingredient approved by the Korean Ministry of Food and Drug Safety. Lycopene, α-carotene, and β-carotene levels were significantly higher in *L. plantarum* HY7712 and HY7715-fermented juices than in non-fermented juice (Figure 2). Additionally, *B. lactis* HY8002 and *L. helveticus* HY7801-inoculated juices had increased levels of lycopene and α-carotene, respectively. The latter juice also displayed a lower lutein content than the control. Tomato pulp fermented with *Lactobacillus sakei*, *Pediococcus acidilactici*, and *Pediococcus pentosaceus* has altered levels of lycopene and β-carotene [73]. *L. plantarum* produces the C30 carotenoid 4,4′-diaponeurosporene [74] and the triterpenoid carotenoid 4,4′-diaponeurosporene. On the other hand, Sanchez-Contreras et al. [75] suggested that lutein might be utilized as a carbon source for the growth of microorganisms. In this study, lutein might also be utilized for the growth of *L. helveticus* HY7801, and we thought that this metabolism in HY7801 could be related to the increase of succinic acid and PI under nutritional depletion in the fermented VJ [48,53]. Consistent with the findings of previous studies, probiotic fermentation of VJ modified its carotenoid content. We hypothesize that the antioxidative effects of elevated carotenoid levels in probiotic VJ are related to the immunity enhancement observed in a mouse model treated with *L. plantarum* HY7712. Orally administered HY7712 restores natural killer cells damaged by γ-irradiation [76] and accelerates the recovery of immunosuppression caused by the anticancer drug cyclophosphamide [77].

## 5. Conclusions

In this study, VJ was fermented with four probiotic strains, *L. plantarum* HY7712, *L. plantarum* HY7715, *L. helveticus* HY7801, and *B. lactis* HY8002, and the metabolite, lipid, and carotenoid content of each juice were analyzed by GC-MS, DI-MS, and LC-MS. The carotenoids, including lycopene, α-carotene, and β-carotene levels, were higher in VJ fermented with *L. plantarum strains* (HY7712 and HY7715) than in the control. Particularly, the levels of uracil and succinic acid were increased in HY7801 fermented VJ, while Proline was also elevated in HY7715. In addition, glycine was increased in VJ fermented with HY8002. We also revealed that a number of metabolic pathways were activated in probiotics during the fermentation process, including the citrate cycle; alanine, aspartate, and glutamate metabolism; glycine, serine, and threonine metabolism; glycolysis; and carotenoid pathways. Compared to previous studies [14,15,16,17,18], we highlighted that three kinds of MS-platforms were applied to analyze many different kinds of metabolites, lipids, and carotenoids of probiotic-fermented VJs, and relative amounts of differentiated biological substances produced by three *Lactobacillus* strains and one *Bifidobacterium* strain were also investigated. Thus, we confirmed that metabolomics and lipidomics are the effective approach to provide more scientific evidence for discovering new beneficial effects of probiotics. Furthermore, we advanced the understanding of how fermented foods mediate their health benefits by revealing the metabolic changes that occur during probiotic fermentation.

## Figures and Tables

**Table 1:** Relative levels of metabolites in the fermented and non-fermented VJs, as detected by GC-MS.

No.	Compound	*m*/*z*	RT (min)	Fragmentation Ions (*m*/*z*)	TMS	Control	*L. plantarum* (HY7712)	*L. plantarum* (HY7715)	*L. helveticus* (HY7801)	*B. lactis* (HY8002)
	**Amino acids**									
1	β-alanine	174	17.37	100, **174**, 248, 290	3	0.352 ± 0.032	ND	ND	0.302 ± 0.011 ^+^	0.328 ± 0.032
2	γ-aminobutanoic acid	174	19.85	**174**, 216, 246, 304	3	83.630 ± 2.806	66.176 ± 8.063 *	84.661 ± 1.777	77.351 ± 2.740 ^+^	81.219 ± 6.131
3	Alanine	116	8.80	100, **116**, 190, 218	2	50.422 ± 1.816	50.480 ± 2.053	50.026 ± 5.861	53.394 ± 3.054	40.845 ± 24.358
4	Asparagine	116	23.13	**116**, 132, 188, 231	3	20.862 ± 1.038	17.253 ± 1.558 *	18.952 ± 0.744 ^#^	13.289 ± 1.015 ^+^	16.524 ± 2.890 ^^^
5	Aspartic acid	232	19.68	100, 202, 218, **232**	3	190.073 ± 8.232	142.789 ± 2.909 *	159.894 ± 1.455 ^#^	157.900 ± 5.864 ^+^	122.123 ± 15.734 ^^^
6	Glutamic acid	246	22.06	128, 156, **246**, 348	3	175.620 ± 4.918	152.488 ± 7.547 *	154.917 ± 3.394 ^#^	134.961 ± 5.251 ^+^	163.626 ± 19.308
7	Glycine	174	14.19	86, **174**, 248, 276	2	3.261 ± 0.147	3.118 ± 0.448	2.453 ± 0.061 ^#^	2.163 ± 0.071 ^+^	5.763 ± 0.237 ^^^
8	Isoleucine	158	13.88	100, **158**, 218, 232	2	6.115 ± 0.241	2.297 ± 0.237 *	1.711 ± 0.171 ^#^	2.861 ± 0.080 ^+^	4.462 ± 1.543
9	Proline	142	13.96	100, **142**, 144, 216	2	4.192 ± 0.234	4.804 ± 0.729	6.941 ± 0.784 ^#^	4.736 ± 0.149 ^+^	3.890 ± 3.297
10	Pyroglutamic acid	156	19.62	133, **156**, 230, 258	2	562.497 ± 15.928	483.337 ± 18.371 *	508.725 ± 11.686 ^#^	521.194 ± 20.262 ^+^	526.650 ± 16.360 ^^^
11	Serine	204	15.68	100, 188, **204**, 218	3	14.700 ± 0.692	1.474 ± 0.232 *	7.357 ± 0.522 ^#^	13.816 ± 0.457 ^+^	10.529 ± 3.635 ^^^
12	Threonine	218	16.32	101, 117, **218**, 291	3	4.661 ± 0.119	1.712 ± 0.157 *	2.217 ± 0.037 ^#^	2.617 ± 0.156 ^+^	4.863 ± 1.161
13	Valine	144	11.80	100, 133, **144**, 218	2	10.732 ± 0.785	5.977 ± 0.323 *	5.387 ± 0.257 ^#^	7.584 ± 0.239 ^+^	7.687 ± 3.507
	**Fatty acids**									
14	1-Monopalmitin	371	45.26	103, 129, 205, **371**	2	4.647 ± 0.403	3.781 ± 1.278	3.705 ± 0.421 ^#^	2.835 ± 0.433 ^+^	4.372 ± 1.145
15	Linoleic acid	75	37.82	67, **75**, 81, 337	1	0.528 ± 0.074	0.394 ± 0.089 *	0.268 ± 0.059 ^#^	0.357 ± 0.044 ^+^	0.337 ± 0.044 ^^^
16	Palmitic acid	117	33.33	**117**, 132, 145, 313	1	0.673 ± 0.049	0.636 ± 0.057	0.649 ± 0.053	0.617 ± 0.090	0.707 ± 0.080
17	Stearic acid	117	38.66	**117**, 132, 145, 341	1	0.240 ± 0.039	0.367 ± 0.083 *	0.286 ± 0.103	0.267 ± 0.081	0.323 ± 0.076
	**Organic acids**									
18	Acetic acid	177	8.12	133, 161, **177**, 205	2	0.143 ± 0.012	0.124 ± 0.008 *	0.137 ± 0.020	0.143 ± 0.016	0.150 ± 0.017
19	Citric acid	273	26.52	**273**, 347, 363, 375	4	120.814 ± 4.491	34.142 ± 1.741 *	50.563 ± 1.841 ^#^	9.052 ± 4.326 ^+^	114.971 ± 4.669
20	Fumaric acid	245	15.46	115, 132, 143, **245**	2	1.181 ± 0.074	0.043 ± 0.005 *	0.045 ± 0.004 ^#^	0.287 ± 0.033 ^+^	0.189 ± 0.036 ^^^
21	Lactic acid	117	7.73	**117**, 133, 191, 219	2	21.595 ± 1.210	602.047 ± 19.653 *	563.702 ± 33.204 ^#^	547.899 ± 14.857 ^+^	420.494 ± 27.212 ^^^
22	Malic acid	233	18.93	133, 189, **233**, 245	3	89.641 ± 5.199	ND	ND	41.678 ± 2.598 ^+^	ND
23	Malonic acid	75	11.51	66, 75, 133, **233**	2	0.441 ± 0.035	0.429 ± 0.023	0.425 ± 0.028	0.419 ± 0.029	0.533 ± 0.115
24	Succinic acid	247	14.48	75, 129, 172, **247**	2	1.544 ± 0.071	1.768 ± 0.071 *	2.462 ± 0.108 ^#^	56.616 ± 3.446 ^+^	3.044 ± 0.131 ^^^
25	Tartaric acid	292	22.43	189, 219, **292**, 423	4	0.776 ± 0.028	0.602 ± 0.084 *	0.638 ± 0.037 ^#^	0.686 ± 0.019 ^+^	0.683 ± 0.034 ^^^
	**Sugars**									
26	Fructose	217	26.34	204, **217**, 319, 437	5	526.553 ± 32.602	553.891 ± 8.751	520.415 ± 26.583	512.824 ± 9.722	521.351 ± 26.811
		103	28.01	**103**, 133, 217, 307	5(MeOX)					
			28.32							
27	Galactose	204	28.89	129, 191, **204**, 217	5	6.029 ± 0.373	5.561 ± 0.336	6.183 ± 0.352	4.300 ± 0.139 ^+^	5.980 ± 0.365
28	Glucose	204	28.61	129, 191, **204**, 217	5	2905.897 ± 138.257	1486.807 ± 39.829 *	2156.601 ± 58.234 ^#^	805.171 ± 102.179 ^+^	2385.734 ± 64.071 ^^^
			31.40							
		319	28.72	160, 205, 217, **319**	5(MeOX)					
29	Glucose-6-phosphate	204	40.26	**204**, 217, 299, 387	6	0.329 ± 0.032	0.288 ± 0.025 *	0.316 ± 0.021	0.251 ± 0.026 ^+^	0.618 ± 0.058 ^^^
			41.74							
30	Sedoheptulose	319	35.74	205, 217, 262, **319**	6(MeOX)	50.748 ± 3.714	42.976 ± 1.234 *	47.967 ± 1.442	49.610 ± 0.918	44.614 ± 3.511 ^^^
			35.89							
31	Sucrose	361	46.06	103, 217, **361**, 437	8	437.362 ± 192.453	556.267 ± 20.170	535.635 ± 35.941	509.509 ± 14.353	507.215 ± 24.494
32	Xylose	103	22.85	**103**, 189, 217, 307	4(MeOX)	0.700 ± 0.042	0.686 ± 0.104	0.708 ± 0.051	0.715 ± 0.047	0.783 ± 0.022 ^^^
	**Sugar acids**									
33	Glyceric acid	189	14.88	103, **189**, 205, 292	3	0.268 ± 0.024	ND	ND	ND	0.148 ± 0.016 ^^^
34	Threonic acid	292	20.66	117, 205, 220, **292**	4	0.569 ± 0.030	0.455 ± 0.031 *	0.502 ± 0.026 ^#^	0.494 ± 0.026 ^+^	0.554 ± 0.029
	**Sugar alcohols**									
35	Erythritol	217	19.22	103, 117, 205, **217**	4	13.869 ± 0.680	1.057 ± 0.045 *	1.119 ± 0.054 ^#^	6.792 ± 0.327 ^+^	1.647 ± 0.436 ^^^
			19.39							
36	Glycerol	205	13.41	103, 117, 133, **205**	3	16.011 ± 0.751	18.614 ± 0.157 *	18.262 ± 0.573 ^#^	16.246 ± 0.480	20.278 ± 0.708 ^^^
37	Mannitol	319	29.66	103, 205, 217, **319**	6	11.188 ± 0.638	9.783 ± 0.434 *	10.961 ± 0.551	10.554 ± 0.242 ^+^	18.955 ± 12.144
38	Myo-Inositol	305	34.45	191, 217, **305**, 318	6	50.657 ± 1.580	45.353 ± 1.026 *	48.372 ± 2.080 ^#^	50.395 ± 1.749	49.615 ± 1.095
39	Xylitol	217	24.25	103, 205, **217**, 307	5	1.149 ± 0.043	1.050 ± 0.050 *	1.122 ± 0.047	1.096 ± 0.051	1.185 ± 0.082
	**Others**									
40	Phosphoric acid	299	13.30	133, 211, **299**, 314	3	262.434 ± 9.607	186.249 ± 12.682 *	201.558 ± 3.812 ^#^	184.653 ± 4.847 ^+^	217.457 ± 7.081 ^^^
41	Uracil	241	15.03	99, 113, **241**, 255	2	0.075 ± 0.011	0.057 ± 0.005 *	0.054 ± 0.006^#^	0.835 ± 0.046 ^+^	0.099 ± 0.064

Mann–Whitney test was performed to detect significant differences between fermented vegetable juices (VJs) and the control. ({*L. plantarum* (HY7712), *; *L. plantarum* (HY7715), #; *L. helveticus* (HY7801), +; *B. lactis* (HY8002), ^}, *p* < 0.05). ND, not detected; RT, retention time; Bold character in fragmentation ions, base peak (the most intensive peak in a GC-MS spectrum); TMS, trimethylsilylation; MeOX, methoxylamine hydrochloride.

**Table 2:** Relative levels of lipids in the fermented and non-fermented VJs, as detected by DI-MS.

No.	Lipid Species	Ion Species	*m*/*z*	Control	*L. plantarum* (HY7712)	*L. plantarum* (HY7715)	*L. helveticus* (HY7801)	*B. lactis* (HY8002)
	**Positive ion mode**							
	Monogalactosyldiacylglycerol (MGDG)							
1	MGDG 18:2/18:3	[M + Na]^+^	799	1.93 ± 0.62	1.74 ± 0.43	1.67 ± 0.47	1.88 ± 0.31	1.55 ± 0.52
2	MGDG 18:2/18:2	[M + Na]^+^	801	14.42 ± 4.00	13.24 ± 3.19	12.68 ± 3.14	13.64 ± 1.71	11.89 ± 3.22
3	MGDG 18:1/18:2	[M + Na]^+^	803	11.51 ± 3.39	10.38 ± 1.90	9.52 ± 1.93	10.37 ± 0.93	9.28 ± 2.10
	Lysophosphatidylcholine (Lyso-PC)							
4	Lyso-PC 18:2	[M + H]^+^	520	5.30 ± 1.04	4.18 ± 1.05	4.36 ± 0.96	4.56 ± 0.70	3.73 ± 0.70 ^^^
5	Lyso-PC 18:1	[M + H]^+^	522	6.56 ± 1.60	6.53 ± 1.16	6.87 ± 1.16	6.38 ± 0.64	6.37 ± 1.01
6	Lyso-PC 22:5	[M + Na]^+^	592	2.81 ± 0.61	2.55 ± 0.52	2.31 ± 0.52	2.80 ± 0.38	2.39 ± 0.56
	Phosphatidylcholine (PC)							
7	PC 18:2/18:2	[M + H]^+^	782	4.29 ± 0.50	4.16 ± 0.23	4.08 ± 0.28	4.06 ± 0.30	4.10 ± 0.30
	Phosphatidylethanolamine (PE)							
8	PE 16:0/20:0	[M + H]^+^	748	1.17 ± 0.30	1.05 ± 0.11	0.98 ± 0.14	1.08 ± 0.11	1.08 ± 0.22
	Triacylglycerol (TG)							
9	TG 16:0/18:2/18:2	[M + NH_4_]^+^	872	3.88 ± 0.26	4.19 ± 0.42	4.13 ± 0.49	4.17 ± 0.59	4.09 ± 0.31
10	TG 18:2/18:2/18:3	[M + NH_4_]^+^	894	2.85 ± 0.19	3.03 ± 0.32	3.01 ± 0.30	3.03 ± 0.39	2.98 ± 0.28
11	TG 18:2/18:2/18:2	[M + NH_4_]^+^	896	9.76 ± 0.61	10.66 ± 1.03	10.45 ± 1.07	10.49 ± 1.31	10.47 ± 0.83
12	TG 18:1/18:2/18:2	[M + NH_4_]^+^	898	3.33 ± 0.18	3.73 ± 0.35	3.68 ± 0.46	3.64 ± 0.56	3.64 ± 0.28
13	TG 18:1/18:1/18:2	[M + NH_4_]^+^	900	1.30 ± 0.04	1.46 ± 0.16	1.46 ± 0.21	1.41 ± 0.20	1.42 ± 0.13
	**Negative ion mode**							
	Phosphatic acid (PA)							
14	PA 16:0/18:2	[M − H]^-^	671	1.81 ± 0.27	1.76 ± 0.21	1.81 ± 0.43	1.84 ± 0.30	1.67 ± 0.13
15	PA 18:2/18:2	[M − H]^-^	695	1.74 ± 0.27	1.56 ± 0.35	1.48 ± 0.35	1.69 ± 0.32	1.44 ± 0.19
	Phosphatidylethanolamine (PE)							
16	PE 16:0/18:2	[M − H]^-^	714	1.46 ± 0.06	1.35 ± 0.08	1.35 ± 0.04 ^#^	1.41 ± 0.09	1.41 ± 0.10
17	PE 18:2/18:2	[M − H]^-^	738	1.23 ± 0.05	3.75 ± 2.19 *	2.72 ± 0.69 ^#^	4.43 ± 2.06 ^+^	1.96 ± 0.31 ^^^
18	PE 18:1/18:2	[M − H]^-^	740	0.73 ± 0.05	1.18 ± 0.83	0.76 ± 0.17	1.15 ± 0.70 ^+^	0.62 ± 0.09 ^^^
19	PE 18:0/18:2	[M − H]^-^	742	4.50 ± 0.84	1.51 ± 0.85 *	1.22 ± 0.49 ^#^	1.01 ± 0.51 ^+^	2.29 ± 0.75 ^^^
	Phosphatidylglycerol (PG)							
20	PG 16:0/18:2	[M − H]^-^	745	1.28 ± 0.12	1.57 ± 0.25 *	1.49 ± 0.30	1.66 ± 0.21 ^+^	1.47 ± 0.20
	Phosphatidylserine (PS)							
21	PS 18:2/20:0	[M − H]^-^	814	0.23 ± 0.04	0.33 ± 0.21	0.21 ± 0.05	0.15 ± 0.08 ^+^	0.27 ± 0.04
22	PS 18:2/22:0	[M − H]^-^	842	0.75 ± 0.12	0.98 ± 0.18 *	0.92 ± 0.09 ^#^	0.91 ± 0.11	0.98 ± 0.14 ^^^
	Phosphatidylinositol (PI)							
23	PI 16:0/18:2	[M − H]^-^	833	6.34 ± 0.86	7.59 ± 1.00	7.44 ± 1.13	8.04 ± 1.21 ^+^	7.50 ± 1.05
24	PI 16:0/18:1	[M − H]^-^	835	1.01 ± 0.15	1.32 ± 0.21 *	1.34 ± 0.23 ^#^	3.58 ± 0.21 ^+^	1.24 ± 0.15 ^^^

Mann–Whitney test was performed to detect significant differences between fermented VJs and the control. ({*L. plantarum* (HY7712), *; *L. plantarum* (HY7715), #; *L. helveticus* (HY7801), +; *B. lactis* (HY8002), ^}, *p* < 0.05).

**Table 3:** Relative levels of carotenoids in the fermented and non-fermented VJs, as detected by LC-MS.

Compound	Formula	RT (min)	*m/z* [M + H] ^+^	Control	*L. plantarum* (HY7712)	*L. plantarum* (HY7715)	*L. helveticus* (HY7801)	*B. lactis* (HY8002)
LUT	C_40_H_56_O_2_	5.61	569.4	4.0 ± 0.2	4.2 ± 0.3	4.2 ± 0.1	2.6 ± 0.5 ^+^	4.4 ± 0.5
LYC	C_40_H_56_	9.32	537.4	30.8 ± 0.9	36.3 ± 0.6 ^*^	45.1 ± 3.0 ^#^	34.0 ± 9.7	38.2 ± 2.1 ^^^
α-CAR	C_40_H_56_	10.95	537.4	50.9 ± 3.5	60.9 ± 5.7 ^*^	69.0 ± 5.9 ^#^	61.8 ± 6.2 ^+^	54.8 ± 4.0
β-CAR	C_40_H_56_	11.12	537.4	113.1 ± 7.6	134.4 ± 13.4 ^*^	147.7 ± 13.5 ^#^	111.3 ± 12.6	120.2 ± 8.7

Mann–Whitney test was performed to detect significant differences between fermented VJs and the control ({*L. plantarum* (HY7712), *; *L. plantarum* (HY7715), #; *L. helveticus* (HY7801), +; *B. lactis* (HY8002), ^}, *p* < 0.05). LUT, lutein; LYC, lycopene; α-CAR, α-carotene; β-CAR, β-carotene; RT, retention time.

**Table 4:** Main metabolic pathways activated in the fermented and non-fermented VJs.

No.	Pathway Name	Compound *^a^*	Total *^b^*	Hits *^c^*	*p ^d^*	Impact *^e^*
1	Alanine, aspartate and glutamate metabolism	alanine, aspartic acid, glutamic acid, asparagine, succinic acid, γ-aminobutanoic acid, fumaric acid	20	7	8.18 × 10^−6^	0.60
2	Glycine, serine and threonine metabolism	glycine, serine, threonine, glyceric acid, aspartic acid	28	5	5.40× 10^−3^	0.42
3	Citrate cycle (TCA cycle)	citric acid, fumaric acid, malic acid, succinic acid	20	4	8.64 × 10^−3^	0.20
4	Aminoacyl-tRNA biosynthesis	asparagine, glycine, aspartic acid, serine, valine, alanine, threonine, proline, glutamic acid, isoleucine	66	10	2.52 × 10^−4^	0.18
5	Starch and sucrose metabolism	fructose, glucose, glucose-6-phosphate, sucrose, xylose	30	5	7.35 × 10^−3^	0.15
6	Arginine and proline metabolism	aspartic acid, fumaric acid, proline, glutamic acid, γ-aminobutanoic acid	40	5	2.47 × 10^−2^	0.10

*^a^* The names of matched compounds from the fermented and non-fermented VJs. *^b^* Total number of compounds in the pathway. *^c^* Number of matched compounds. *^d^* Original *p* value calculated from the uploaded data. *^e^* Pathway impact value calculated from pathway topology analysis.

**Table 5:** Metabolites and lipids with VIP values > 1.0 in the PLS-DA model.

No.	Compound	VIP Value
1	γ-aminobutanoic acid	1.71
2	Glycine	1.51
3	Glucose-6-phosphate	1.43
4	Uracil	1.42
5	β-alanine	1.42
6	Succinic acid	1.41
7	Linoleic acid	1.41
8	Aspartic acid	1.39
9	Galactose	1.38
10	Phosphatidylinositol (PI) 16:0/18:1	1.36
11	Proline	1.35
12	Asparagine	1.33
13	Serine	1.29
14	Glycerol	1.28
15	Sedoheptulose	1.23
16	Glucose	1.23
17	Myo-inositol	1.20
18	Malic acid	1.16
19	Erythritol	1.14
20	Isoleucine	1.13
21	Threonine	1.13
22	Fructose	1.12
23	Fumaric acid	1.08
24	Citric acid	1.08
25	Glyceric acid	1.03
26	Malonic acid	1.03
27	Xylitol	1.01
28	Phosphatidylethanolamine (PE) 18:0/18:2	1.00
29	Glutamic acid	1.00
